# Systematic Construction of Kinetic Models from Genome-Scale Metabolic Networks

**DOI:** 10.1371/journal.pone.0079195

**Published:** 2013-11-14

**Authors:** Natalie J. Stanford, Timo Lubitz, Kieran Smallbone, Edda Klipp, Pedro Mendes, Wolfram Liebermeister

**Affiliations:** 1 School of Computer Science, Manchester Centre for Integrative Systems Biology, University of Manchester, Manchester, United Kingdom; 2 Institut für Biologie, Theoretische Biophysik, Humboldt-Universität zu Berlin, Berlin, Germany; 3 School of Computer Science, Manchester Centre for Integrative Systems Biology, University of Manchester, Manchester, United Kingdom; 4 Institut für Biologie, Theoretische Biophysik, Humboldt-Universität zu Berlin, Berlin, Germany; 5 School of Computer Science, Manchester Centre for Integrative Systems Biology, University of Manchester, Manchester, United Kingdom; 6 Virginia Bioinformatics Institute, Virginia Tech, Blacksburg, Virginia, United States of America; 7 Institut für Biochemie, Charité - Universitätsmedizin Berlin, Berlin, Germany; University of Erlangen-Nuremberg, Germany

## Abstract

The quantitative effects of environmental and genetic perturbations on metabolism can be studied *in silico* using kinetic models. We present a strategy for large-scale model construction based on a logical layering of data such as reaction fluxes, metabolite concentrations, and kinetic constants. The resulting models contain realistic standard rate laws and plausible parameters, adhere to the laws of thermodynamics, and reproduce a predefined steady state. These features have not been simultaneously achieved by previous workflows. We demonstrate the advantages and limitations of the workflow by translating the yeast consensus metabolic network into a kinetic model. Despite crudely selected data, the model shows realistic control behaviour, a stable dynamic, and realistic response to perturbations in extracellular glucose concentrations. The paper concludes by outlining how new data can continuously be fed into the workflow and how iterative model building can assist in directing experiments.

## Introduction

Improving understanding of metabolic behaviour is vital to drive research outcomes in medical and biotechnology fields. Building mathematical models of metabolism, whether as a whole, or as constituent pathways, allows the behaviour to be comprehensively investigated in order to generate hypotheses which can be tested in the laboratory. Metabolic models show a loose dichotomy, containing either a few reactions described to high kinetic detail (“kinetic models”), or a large set of reactions with little or no kinetic detail at all (“stoichiometric” or “constraint-based” models). For the well-studied model organism *Saccharomyces cerevisiae* (baker’s yeast), many kinetic models are available in BioModels Database [1]. Whilst these models allow for dynamic simulations and control analysis, their coverage does not extend far beyond the glycolytic pathway: they typically contain less than 20 reactions, which is, by far, not enough to understand the global dynamics of metabolism. Larger models such as the yeast consensus model [2] contain more than 1000 reactions, but merely define the stoichiometry of the metabolic network [3], which can only be studied using techniques such as Elementary Mode Analysis [4] or Flux Balance Analysis (FBA) [5]. The lack of kinetic detail in larger models results from a dearth of data: the “data-deluge”, from a kinetic perspective, never really happened [6]. Ways to circumvent the lack of data, by looking at network reaction control under parameter uncertainty, have been developed [7]–[10], but these techniques do not provide explicit kinetic solutions to the system.

Construction of large dynamic and kinetic models have been attempted using both constraint-based, and kinetic research paradigms. Constraint-based approaches, including dynamic FBA [11] and structural kinetic modelling [12], [13], start from stationary fluxes and introduce pseudo-kinetic behaviour. Conversely, kinetic models contain rate laws determined reaction by reaction (derived either experimentally or from the literature), and then combined [14], an example being Teusink’s glycolysis model, built using detailed *in vitro* kinetics [15]. Larger models, containing many reactions with undetermined rate laws, must be built using standard rate laws [16]–[20] and methods for completing the missing parameter data. Smallbone (2010) [21] exemplified one of the first large-scale kinetic constructions, populating the stoichiometric yeast consensus model with linlog rate laws and using a previously developed approximation methodology [22]. The methodology uses knowledge of the stoichiometric matrix, and flux balance analysis, to generate data covering a large network. Following this, Li (2010) [23] developed a workflow that uses a known metabolic network and inserts kinetic rate laws from Sabio-RK [24]. Where suitable rate laws are not available, a generic rate law is inserted instead. The parameter values are obtained from various databases where possible. These methods exemplify the main steps for constructing such a model: (i) the stoichiometric network must be reconstructed, (ii) kinetic rate laws need to be assigned to all reactions, and (iii) the kinetic constants in the rate laws must be determined.

Current methodologies, however, do not go far enough towards generating a model of the calibre required for the current needs. Large kinetic models would have to show both a steady state with realistic metabolic fluxes and concentrations, and a consistent equilibrium state. This imposes a variety of constraints on the model parameters, and naive constraint-based or kinetic approaches do not satisfy these requirements. Parameter balancing [25] solves this problem: in place of inserting reported kinetic constants directly into the model, it uses them as input data, translates them into a consistent parameter set, and ensures that all thermodynamic constraints are satisfied. Parameter balancing, however, is unable to process metabolic fluxes or rate equations directly: all of which are vital for constructing a usable model. We therefore combined approximation techniques with flux analysis, thermodynamic analysis, and parameter balancing into a new methodology that can create consistent models in realistic biological states. A key aspect of this method is the approximation techniques which allow small, incomplete data sets to be used in order to generate kinetic data for a whole model. This helps circumvent many construction issues associated with a lack of available data.

The model produced from the method is not a final model, and its falsifiability is its strength. To quote Gutenkunst *et al* (2007) [26]:

“For computational modeling to be useful in incompletely understood systems, we must focus not on building the final, perfect, model with all parameters precisely determined, but on building incomplete, tentative, and falsifiable models in the most expressive and predictive fashion feasible”.

Where the model does not predict experimental outcomes, despite inclusion of relevant data, it forms a basis for asking “why?”. This will lead to chances of obtaining more insight through top-down analyses of ‘omics data, and then modifying experiments to obtain the most useful data for improving the model, something Heinemann *et al* (2010) [27] suggest is a vital aspect of systems biology research. The steps of the method can easily be repeated, allowing an iterative model improvement, which in turn should lead to a model that is more of a “final” representation of the metabolic behaviour of the organism.

## Results

### Workflow for Model Construction

**Figure 1:**
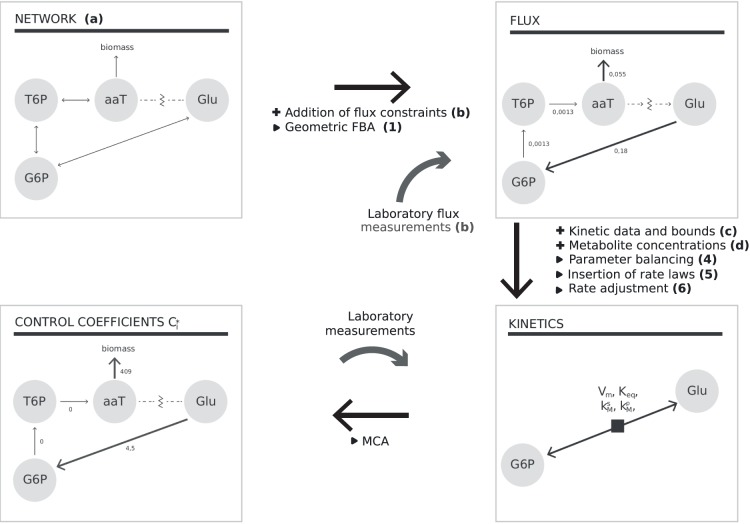
Stages of data addition (+) and method runs ([>]) in the workflow. Letters and numbers in parentheses refer, respectively, to input data and to the stages of the workflow. (1) Geometric FBA [32] is run, yielding a thermodynamically feasible, stationary flux distribution matching the flux data; (2) a sub-network of flux-carrying reactions is exported, (3) consistent values of all metabolite concentrations and equilibrium constants are determined, (4) kinetic constants appearing in the sub-network are balanced, (5) kinetic rate laws and associated kinetic constants are inserted into the model, and (6) the maximal reaction rates are adjusted to reproduce the steady-state fluxes calculated before. The laboratory data feeding into “Flux” can include quantitative metabolomic measurements of metabolites, and also dynamically calculated flux values for the network. The lower half of the diagram shows a cycle of experimentation and modelling: here the result of the MCA can be used to target which rate laws should be measured *in vitro* after measurement, this rate law can be substituted into the model, with a view to better fit the observed perturbation behaviour. All grey arrows refer to aspects of the workflow where additional data can be added in as knowledge increases. The pathway shown is a truncated version of the trehalose pathway. Abbreviations: T6P = trehalose 6-phosphate; aaT = 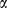-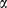 trehalase; Glu = Glucose; G6P = glucose 6-phosphate.

We developed a workflow for systematically converting metabolic reconstructions into large-scale kinetic models of metabolism (see Fig. 1). It is designed to take sparse or full data sets, performs a thorough analysis of parameter constraints, and then generates the kinetic model using massive data integration. In particular, it addresses steps (ii) and (iii) of model construction, and their associated issues. The resulting models meet the following requirements: the kinetic constants are close to measured values or, where data are not available, in biochemically plausible ranges; the model reproduces a steady state with realistic stationary fluxes and concentrations; model parameters and stationary fluxes are consistent with thermodynamic laws, ensuring the existence of a consistent equilibrium state.

**Figure 2:**
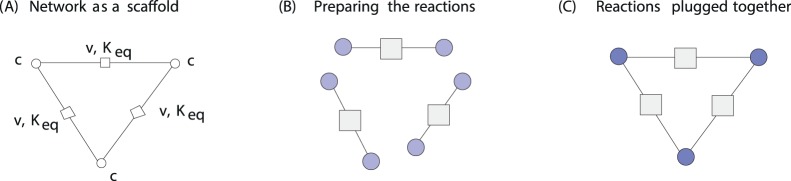
Consistent parameter sets for large kinetic models. (A) The metabolic network provides a frame for formulating parameter dependencies. Stationary fluxes 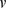, concentrations *c*, and equilibrium constants 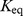 must be thermodynamically consistent. (B) For each reaction, the Michaelis constants 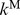 and 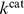 need to agree with the predefined quantities 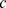, 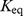, and 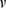. (C) Given a consistent parameter set, enzymatic reactions can be safely connected. The resulting model will actualise a predefined steady state, with rate laws satisfying the following conditions: (i) Quantities shared by several reactions – for instance, metabolite concentrations – have the same values in each of them. (ii) For internal metabolites, incoming and outgoing fluxes are balanced. (iii) Quantities that arise from differences along reactions satisfy the Wegscheider conditions: for instance, their sums over closed loops vanish. (iv) The kinetic constants satisfy the Haldane relationships, which relate the kinetic constants of a rate law to the equilibrium constant of the reaction. (v) Flux directions agree with thermodynamic forces, given by the negative differences 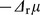 of chemical potentials.

We use the metabolic network as a frame in which certain quantities are predefined (see Fig. 2). All quantities involved in more than one reaction – fluxes, metabolite concentrations, and equilibrium constants – are chosen in agreement with the network constraints. Then, consistent Michaelis constants and catalytic constants are selected, for every reaction, using parameter balancing. The resulting model parameters satisfy all Wegscheider conditions [28], Haldane relationships [29], and observed flux directions, and after a simple rescaling of enzyme levels, actualise the predefined steady state. Enzyme re-scaling is based on strategies outlined in Kholodenko *et al* (1998) [30]. The resulting models could immediately be used to direct experimentation and accurately predict certain biological behaviours. The workflow is extendable to all organisms for which there is a genome-scale metabolic reconstruction established (something which is becoming more accessible for many organisms [31]), and which can be cultured under steady-state conditions. This addresses the needs of systems biology and biotechnology by bridging the void between kinetic models, stoichiometric models, and experimental investigation.

The basic workflow and associated methods are illustrated in Fig. 1, where letters represent data input or output and numbers represent methods to process the data. First, we shall introduce each of the pieces of data and methods contained within the workflow and explain them in a general way. Then, we present a kinetic model of yeast metabolism, built using this workflow, and discuss some of its dynamic and control properties. The model construction is described in detail in the Methods section.

#### Data used in the workflow

The workflow allows for a seamless integration between measured data and data calculated using approximation methodologies. Numerical values are not directly inserted, but adjusted in order to produce a biologically feasible model that obeys the physical laws. As shown in Fig. 1, the input data include (a) a network structure, (b) flux data, (c) kinetic and thermodynamic constants, and (d) metabolite concentrations. The network structure defines the stoichiometric model of an organism’s metabolism, which can be large. It exhaustively lists the reactions to appear in the final network, including all reactants and products. Allosteric effectors can be included if known, and isoenzymes can also be maintained in the network. The flux data comprise a non-exhaustive set of reaction fluxes for a single stationary state, and for the growth conditions in question. Some reaction fluxes may be set to 0 to reflect details of the strain (e.g. gene knockouts), and transport fluxes across the outer cell membrane may reflect nutrient availability in the growth medium used in the laboratory. The flux data may be obtained from detailed kinetic models at the desired steady state, 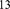
C measurements, or a combination of both. The metabolite concentrations need not be an exhaustive list and can either be taken from measurements, from existing kinetic models, or can be assigned based on knowledge of the extracellular medium, or a combination of all. For metabolites of unknown concentration, the median value of all known concentrations is accepted as a default concentration. The kinetic and thermodynamic constants can include a non-exhaustive list of equilibrium constants and kinetic constants such as catalytic constants or Michaelis constants, which should ideally be known for the conditions the strain is grown at.

#### Steps of the workflow

The workflow consists of a series of methods, allowing for a layered construction of a kinetic model holding the desired steady state (see Fig. 1). First, Geometric FBA [32] is used to determine a thermodynamically feasible, stationary flux distribution matching the flux data (for a feasibility test, see Note ii in File S1). Then, the network is reduced to a sub-network of active, i.e. flux-carrying reactions. The criterion for reducing the network can also be modified (i.e. by setting a minimum flux cut-off point), or omitted (thus retaining the full network) depending on the purpose of model construction (see Note i in File S1). Next, equilibrium constants and metabolite concentrations, consistent with the flux directions, need to be determined. As a pragmatic approach, the predetermined metabolite concentrations are held fixed and the equilibrium constants are adjusted within constraints. A number of known equilibrium constants (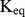) are used as fixed constraints on the system, while unknown equilibrium constants are calculated using linear regression to least squares, such that the Wegscheider conditions 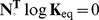 are satisfied (where 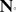 is a kernel matrix of the stoichiometric matrix). The associated mass-action ratio 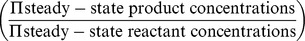 +10% is set as the lower bound, and 1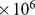 is set as the upper bound. At this point, we have obtained a complete and thermodynamically consistent set of fluxes, concentrations, and equilibrium constants. Experimental data and bounds on kinetic constants are now used, in conjunction with parameter balancing [25], to obtain a complete set of kinetic constants that obey both Wegscheider conditions and Haldane relationships. The rate laws, with the balanced parameters, are then inserted into the network. The resulting kinetic model, with the chosen metabolite concentrations, show the right flux directions, but not the correct steady-state fluxes as determined in step 1. Thus, all maximal velocities 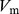 are adjusted to make the reaction rates reflect the desired steady state.

### Large-scale Yeast Model

**Figure 3:**
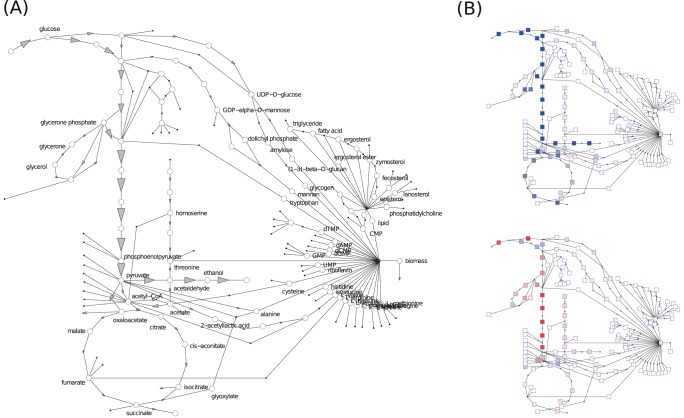
Fluxes and control coefficients in the yeast metabolic model. (A) Fluxes obtained from Geometric FBA. Only selected reactions with large fluxes are depicted, co-substrates are not shown (flux directions and magnitudes shown by arrows). (B) Flux control coefficients. Top: Control exerted by the glucose transporter (GluT). Unscaled flux control coefficients are shown in shades of blue (positive values) and red (negative values). Bottom: control exerted by the biomass production reaction. High-flux reactions respond most strongly: an increased glucose import increases the glycolytic flux, while increased biomass production directs fluxes to other pathways and thereby decreases the glycolytic flux. Flux control coefficients for a model with allosteric regulation are shown in Figures G and H in File S1.

As an example case for our workflow, we generated a large-scale kinetic model of yeast metabolism. *Sacharomyces cerevisiae* is one of the most studied eukaryotic model organisms, and a comparatively large amount of data is available for fluxes, concentrations, and equilibrium constants. The model generation process is described in detail in the Methods section. The final model contains 285 flux-carrying reactions and 294 metabolites. As expected, it displays a large flux through the glycolytic pathway and a large production of ethanol (see Fig. 3 (b)). This behaviour is primarily defined by the flux input from the kinetic models used and reflects the expected behaviour when growing the organism in the laboratory. A detailed list of flux values is given in S4.

Being able to direct the model into the desired steady state is a significant improvement over previous methods [21], [23] and to the basic implementation of parameter balancing [25]. However, our construction does not guarantee that the steady state is stable. A model in a stable steady state will return to that state after a small perturbation in a system variable, whilst a model with an unstable steady state, when perturbed, will show increasing divergence or oscillatory behaviour and is therefore fragile against molecular noise. In this instance, the model demonstrates a stable steady state with strictly negative Jacobian eigenvalues. We confirmed this by perturbing the model using a ten-fold increase of extracellular glucose and mapping the relaxation of the system back to the original state. The stability is a clear improvement over the large-scale model presented in Smallbone 2007 [22], which showed an unstable steady state with 17% of the eigenvalues having a positive real part, the largest value being around 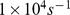. We believe that ensuring thermodynamic consistency and introducing the saturable rate laws have been key contributors to the stability of the system.

To study the model response to changes in enzyme concentrations, we performed metabolic control analysis (MCA, see Methods section for details). The resulting control coefficients describe the relative flux or concentration changes caused by relative changes in enzyme levels. The results in Fig. 3 show that overall flux control (see Methods) is heavily dominated by reactions that balance and produce co-factors. In addition, high control is exerted by some early reactions of the citric acid cycle. A study by Thomas and Fell (1998) [33] demonstrates that ATP-utilising reactions show a high control over flux changes within glycolysis. However, transport reactions also show high control over metabolic fluxes in a number of organisms [34]–[36]. Others have shown that the control is distributed between ATP-utilising reactions and glucose transport [37]. Whilst there is literature to support the control patterns we have identified, more experimental data and evidence will have to be collected to be certain of the accuracy.

We compared the flux control in glycolytic enzymes of the large yeast model with the flux control in each of the small yeast models used to construct it (see Table 1). Even though the large model contains input data from the smaller models, we expected to see changes in the control distribution because control is a systemic property and will be affected by extending the system. Within the large model, linear chains of reactions from glycolysis are now involved in branch-points between other major pathways, including pentose phosphate pathway, trehalose biosynthesis, and the citric acid cycle. This has led to a clear shift in the control distribution in glycolysis.

**Table 1:** Overall flux control exerted by different enzymes in glycolysis.

	Large model	reg. model	BM: 61	BM: 64	BM:172	BM:176	BM:177
Highest 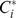	ADH	ATPase	HXK	GluT	GluT	GluT	GluT
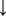	ATPase	G3PDH	ADH	HXK	HXK	HXK	HXK
	Eno	HXK	ATPase	G3PDH	G3PDH	G3PDH	ATPase
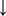	FBPA	ADH	PFK	ATPase	ATPase	ATPase	G3PDH
	GluT	GAL3PD	GAL3PD	GAL3PD	GAL3PD	ADH	ADH
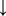	GAL3PD	PGK	G3PDH	ADH	ADH	Eno	Eno
	G3PDH	PGM	GluT	Eno	Eno	PFK	PFK
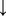	HXK	Eno	–	FBPA	PGK	FBPA	PGK
	PFK	PFK	–	PGK	PFK	PGK	FBPA
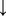	PGK	FBPA	–	PFK	PGM	–	PGM
Lowest 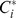	PGM	GluT	–	PGM	FBPA	–	–

The general flux control exerted by a reaction is quantified by 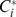, the sum of squared scaled control coefficients. Control coefficients were calculated at the operating state from the large-scale yeast model and from the five original models*^a^* used to define the flux and concentration values. Only a selection of enzymes appearing in the original models are shown*^b^*.

a Model references are as follows BM:61 [43], BM:64 [44], BM:172 [45], BM:176 [46], and BM:177 [46]

b Abbreviations as follows: ADH, alcohol dehydrogenase, reverse reaction; ATPase, cytosolic ATPase; Eno, enolase; FBPA, fructose-bisphosphate aldolase; G3PDH, glycerol-3-phosphate dehydrogenase; GAL3PD, glyceraldehyde-3-phosphate dehydrogenase; GluT, glucose transport; HXK, hexokinase; PFK, phosphofructokinase; PGK, phosphoglycerate kinase; PGM, phosphoglycerate mutase.

Many of the models used to construct the large model contained enzymatic regulation by allosteric effectors. To ensure that the shift in control behaviour was not due to the lack of regulation in the large model, we generated a version of the model with known regulation included. Whilst there were no dramatic changes in the pattern of overall control, the control within the glycolytic pathway was largely shifted towards trends seen within the smaller models. High control was observed for ATPase, glycerol-3-phosphate dehydrogenase, hexokinase, alcohol dehydrogenase, and glyceraldehyde-3-phosphate dehydrogenase. Low control was seen for phosphoglycerate kinase, phosphoglycerate mutase, enolase, phosphofructokinase, and fructose bisphosphate aldolase. The control for glucose transport decreased further, showing the lowest control out of all the enzymes in the pathway under conditions where regulation is included. We also noted that the model with allosteric regulation is not stable at the constructed steady state and, when integrated, moves to a close-by stable steady state. We believe this is related to the resolution of the steady state calculation being set to 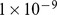 therefore small numerical differences during the rate scaling, particularly in the smaller fluxes (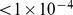), will cause perturbations away from the scaled state. The changes caused by allosteric regulation are interesting to note because our knowledge of systemic enzymatic regulation is poor. Given these findings, we expect the control distribution from the model to be subject to major changes when new regulation is discovered and included.

Using a 30% increase in extracellular glucose, we simulated the dynamic response to perturbations in both the standard model and the model including regulation. All plots can be seen in Figures A–F in File S1. Both the standard model and the regulation model demonstrate very similar behaviour during the perturbation. Both models qualitatively reproduced expected behaviours such as increase in extracellular glucose causing increased flux in glucose transport, glucose-6-phosphate isomerase, and ethanol transport, pushing the model towards increased fermentative metabolism. There is also a small increase in biomass production. The converse behaviours are true during decreased levels of extracellular glucose.

The changes in metabolite concentrations around key metabolite pools, where flux is diverted into different pathways, also primarily conform to what we would expect: there is a general increase in concentrations leading through to fermentation. Amino acid pools show a short delay before they respond to the changes in extracellular glucose. Since it takes some time for the increased glucose to proliferate through the interim metabolic pools, this is something we would expect, unless there are regulators that alter biomass production to respond to changes in extracellular glucose more rapidly. Co-factor balances within the model generally behave as expected, showing a conservation between their concentrations. We noted that ATP concentration decreased upon addition of extracellular glucose. This seemed counter-intuitive, given that ATP regeneration depends on energy provided by glucose, but this paradoxical phenomenon has been observed in laboratory as a normal physiological behaviour of the cell [38], [39]. It is demonstrative of the turbo effect where ATP is first rapidly consumed in upper glycolysis, leaving a lag before it is later produced through aerobic metabolism. This behaviour is not captured by the small models, which were used in contraction of the large model. It is therefore an emergent dynamic property of the large-scale system, and highlights the utility and need for creating large kinetic models.

## Discussion

In the framework described, models are constructed from all available data, and knowledge gaps are filled based on reasonable assumptions about biochemical constants and variables, and on the many constraints between them. A general concern in kinetic modelling is whether model parameters can be reliably determined from the available data. Ideally, a model should fit the data and, at the same time, the data should suffice to determine the model parameters. In kinetic models with incomplete data, gathered from different sources, both requirements will inevitably be compromised. While imperfect model fits are typically treated by least-squares fitting, non-identifiable parameters can either be assessed by analysing the model structure or be handled by regularisation, i.e. heuristic rules for picking one out of the many possible parameter sets. Bayesian parameter estimation is located in between: formally, the use of non-uniform priors makes all parameters – i.e., their posterior modes – identifiable, but parameters that are not restricted by data may still show large posterior uncertainties.

Both approaches, regularisation and Bayesian estimation, play a role in our model construction. On the one hand, Geometric FBA effectively uses regularisation to determine the flux distribution: enforcing small fluxes will, in general, make the flux distribution identifiable; of course, other heuristics could be used instead. On the other hand, parameter balancing yields a joint posterior distribution of the kinetic constants, entailing uncertainty ranges for individual parameters and correlations between them. The remaining uncertainties can be assessed by studying the posterior distribution, and parameters that remain poorly determined can be easily spotted by their large uncertainty ranges.

To construct and assess a whole range of possible models, feasible flux distributions could be sampled and kinetic constants could be drawn from their posterior distribution, leading to an ensemble of kinetic models, all matching the input data. By selecting models with stable steady states, simulating many of them, and assessing their dynamic properties, one could obtain probabilistic statements about model dynamics and control.

Over time, comprehensive cell models will have an impact on biotechnology and medicine, owing to the reduced time and costs associated with a more targeted approach to experimentation. We have demonstrated the use of the method to generate a model with and without known allosteric regulation, and the method can be applied to any organism with an available stoichiometric reconstruction, and which can be cultured under constant conditions in the laboratory. Even imperfect data can be useful in model construction, and as more is known about the organism being researched, the modelling results can be iteratively improved.

Many of the constants and concentrations within the model are computed using approximation values. These estimations could be considerably improved by taking experimental values for all parameters. However, it will take some time before large-metabolic coverage of such data becomes available. In a natural extension to the methodology, data collection could be targeted using the method proposed in Grimbs (2007) [13] where MCA is used to determine reactions with high flux control, which would be candidates for model refinement: for instance, rate constants could be measured *in vitro* and be substituted into the workflow. With iterative steps, the model is expected to show increased accuracy in predicting the transient behaviour of the system during small perturbations.

Our method is a helpful complement to current interdisciplinary approaches, where modelling and experimentation are closely weaved and each informs the other. Through iterative cycles, the model will become more comprehensive and prove extendable for more complex investigations into cellular behaviour, such as metabolic re-routing, the effects of knockouts, and growth responses to changes in the environment.

To ensure that readers can successfully implement the workflow and adapt it for their purposes, we provide an in-depth range of considerations for generating the model in Note i in File S1. In particular, any method to determine thermodynamically feasible fluxes, as well as associated equilibrium constants and metabolite concentrations, could be used to replace the first steps of our workflow. We recommend that the workflow is followed by a dedicated modeller due to the specialised methods, and potential adaptions, required throughout. The workflow should also be supplemented by cross-discussion with laboratory scientists to ensure that the models represent the known biology of the organism as correctly as possible.

## Materials and Methods

### Tools and File Formats

To generate a large-scale kinetic model of yeast metabolism, a set of tools and scripts were used to process different file formats. For the model itself, the standard format SBML (Systems Biology Markup Language [40]) was used throughout the workflow. Flux data and metabolite concentrations were integrated into the process via normal spreadsheets, whilst the parameter list for parameter balancing was given in the table format SBtab (Systems Biology Table, http://www.sbtab.net). MATLAB and COPASI [41] were used for numerical analyses and data generation. The software for parameter balancing is written in Python [42] and available from SourceForge (https://sourceforge.net/projects/parbalancing). The final SBML models produced for yeast can be found in BioModels database (http://www.ebi.ac.uk/biomodels/). The original model is MODEL1204270000 (see File S2), and the regulation model is MODEL1307040000 (see File S3).

### Network [Data a]

The Yeast 4.0 model [3] was used to define the stoichiometric matrix. This is a comprehensive reconstruction of the yeast metabolism, which demonstrates a large improvement on lipid metabolism compared to previous reconstructions, and also shows a higher connectivity between the reactions.

### Flux Data [Data b]

To obtain a set of flux data, we selected a group of metabolic models from BioModels Database [1] that are yeast-specific and contain glucose as the primary carbon source (models 61 [43], 64 [44], 172 [45], 176 [46], and 177 [46]; see Supporting Table 2 in File S1). Each model was run to steady state from its operating state and the resulting flux for each reaction was noted. Where more than one model provided flux values for the same reaction, the median value was used. This provides an approximation of the flux when yeast is grown using extracellular glucose as the sole carbon source. The directions of allowed flux through the transport reactions were kept as described in the original Yeast 4.0 model.

### Flux Balance Analysis [Step 1]

A flux distribution was computed by Geometric FBA [32], an algorithm that iteratively reduces the solution space based on the principle of minimal sum of fluxes. The resulting fluxes are at steady state and thermodynamically feasible, unless the imposed flux constraints force futile cycles, and should yield a more biologically realistic result because of the extra constraints used for computation. The flux data (see Data b) were used to constrain the lower and upper flux bounds of the associated reactions during Geometric FBA calculation. The Geometric FBA solution has to adhere as closely as possible to the constrained fluxes, whilst also satisfying the chosen objective function of the system, which was to maximise growth. The confidence in a flux value can be expressed by allowing smaller or larger upper and lower bounds around the given value. Larger bounds should be allowed for reactions showing a significant change in pathway position (i.e. when a reaction previously located in a linear chain is now a branching point).

### Export Network of Interest [Step 2]

From the resulting flux distribution, we removed all reactions with vanishing fluxes. The pathways represented within the model can be seen in Supporting Table 1 in File S1. Reactions with negative fluxes were reversed with respect to sign of rate and stoichiometric coefficients to produce positive fluxes for all reactions. The reversal is not strictly necessary but simplifies the computations used in further steps of the workflow. This step reduced the network to a central subnetwork of flux-carrying reactions. As more data are obtained, the network can be expanded. To further reduce the network in size, reactions may also be omitted using a finite cut-off value for the fluxes (e.g. 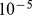 mM/s). In these instances, the Geometric FBA needs to be rerun, using the reduced network as an input, to ensure there is still a solution to the system. The reduced flux solution should also be retested for thermodynamic feasibility, which is vital to ensure that the flux scaling in Step [5] will be possibly.

### Kinetic and Thermodynamic Constants [Data c]

Where appropriate, the equilibrium constants were taken from models available in BioModels Database that use glucose as their primary carbon source (BioModels 61 [43], 64 [44], 172 [45], 176 [46], and 177 [46]; see Supporting Table 5 in File S1). These values were used as fixed data points. All transport reactions were set to have equilibrium constants of 1. The Michaelis constants were initially assigned values equal to the steady-state concentration of their corresponding metabolite. This helped generate sensible upper and lower constraint bounds, these were used during parameter balancing. Constants for allosteric regulators were taken as averages of those found in the BRENDA database [47], the values used can be found in Supporting Table 7 in File S1.

### Metabolite Concentrations [Data d]

Intracellular and extracellular metabolite concentrations were taken from yeast-specific models in BioModels Database that use glucose as the primary carbon source (see Supporting Table 3 in File S1). Unknown intracellular concentrations were set equal to the median value of known intracellular concentrations, 0.549 mM. These are listed in Supporting Table 3 in File S1. For the extracellular concentrations, the median value of all extracellular concentrations in Supporting Table 4 in File S1 was used (24.5 mM). Some extracellular concentrations were manually adjusted (see Supporting Table 6 in File S1)). Protons and phosphate were assumed to have fixed concentrations within the system.

#### Calculating consistent equilibrium constants [Step 3]

A full set of equilibrium constants was computed for addition into the model using the method outlined in parameter balancing. Unknown constants were allowed to vary between 10% above the reaction’s mass action ratio and an upper bound of 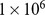. To prevent violation of the thermodynamic laws, the Wegscheider conditions for equilibrium constants must be satisfied. For instance, in a network consisting of a simple closed cycle, multiplying all equilibrium constants must yield a value of 1. We used a least-squares approach to parameterise the equilibrium constants such that the following Wegscheider conditions were satisfied:
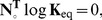
(1)where 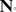 is a null space matrix of the stoichiometric matrix, containing a complete set of stationary flux vectors as its columns, and 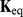 is the vector of equilibrium constants.

### Balancing the Kinetic Constants [Step 4]

For creating a complete and feasible set of kinetic constants and their uncertainty ranges, we used parameter balancing [25]. The kinetic constants are mutually dependent due to thermodynamic constraints (e.g. the ratio of catalytic constants of a reaction can be calculated from the equilibrium constant and the Michaelis constants through the Haldane relationship). Thus, on the one hand, the parameter set can be complemented for missing values. On the other, different data values may be in conflict and require adjustment. Parameter balancing exploits these dependencies and searches for a most plausible set of kinetic constants, based on Bayesian parameter estimation and on the current and limited collection of kinetic constants available. To resolve underdetermined values, it uses prior distributions of every type of parameter (such as Michaelis constants or catalytic rate constants; for the exact values, see [25]). Since independent priors can capture only some types of kinetic constants (the mutually independent ones), we use pseudo values [13], in addition, to represent prior knowledge on other, dependent kinetic constants. Although priors and pseudo values are provided with large standard variations, they can still raise the reliability of the estimate in comparison to not using any values at all. Eventually, because of the constraints between parameters, the estimate of a parameter – e.g. some Michaelis constant in the model – will not only depend on data for this Michaelis constant, or on the prior of Michaelis constants in general, but also on known equilibrium constants, catalytic constants, and their priors. The estimation is performed within a Bayesian framework and results in a posterior distribution, from which the mean and median values - as well as the corresponding standard deviations - can be obtained as point estimates for each of the kinetic constants.

The central step in parameter balancing, finding the posterior mode of the kinetic constants vector within predefined boundaries is a convex optimisation problem, so gradient-based optimisation algorithms can be used. Nevertheless, since all parameters of a model are coupled by constraints, the calculation can become demanding for larger networks. To reduce the computational effort, we broke down the network into single reactions and balanced the Michaelis constants and catalytic constants for each of them individually. As shown in Fig. 2, a network can be split into single reactions and plugged together again, without losing its thermodynamic feasibility, but only if the shared quantities are held fixed. Therefore, the equilibrium constants needed to be predetermined (in Step [3]) and accepted as fixed values during parameter balancing.

### Assigning Kinetic Rate Laws [Step 5]

The values obtained from parameter balancing can be used as kinetic constants in the rate laws of our model. For this task, we chose the common modular rate law [20], a generalised form of the reversible Michaelis-Menten kinetics applicable to any reaction stoichiometry. Regarding a reaction 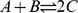, for instance, we can calculate the rate 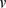 in mM/s as
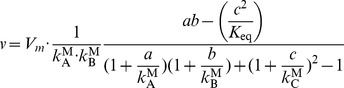
(2)where


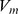
 = maximal reaction velocity    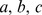 = concentrations (mM)


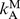
 = dissociation constant of A    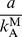 = reactant constants of A


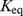
 = equilibrium constant

This rate law represents a random-order enzyme mechanism, the terms of the denominator, when expanded, each represent one possible binding state of the enzyme. The common modular rate law resembles the convenience kinetics [18] with only slight modifications. As an exception, the rate laws for biomass, growth, and lipid production reactions (all operating at the same flux rate) were replaced by linlog rate laws, or, whenever the rate law yielded negative values, by a value of 0 (i.e. a rate law akin to 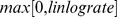).

### Adjusting the Maximal Velocities to Steady-state Fluxes [Step 6]

The desired flux through each reaction at steady state had already been calculated in Step [1], and the later steps of the methodology have ensured that the directions of each reaction rate is the same as the pre-calculated fluxes. Therefore, to match the rates to the steady-state fluxes, the maximal velocities 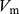 can simply be rescaled by positive factors. This ensures that the model is at steady state at the point of construction.

### Metabolic Control Analysis (MCA)

Metabolic control analysis is a technique for studying how system variables such as fluxes and concentrations are affected by small changes in system parameters. It uses control coefficients, of which there are two types: flux control coefficients 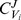 and concentration control coefficients 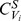. They measure how local perturbations of reaction rates affect the steady-state flux (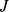) or steady-state concentration (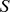) of the system and are defined as follows:
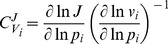
(3)

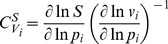
(4)where 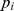 is a parameter affecting exclusively the 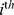 reaction, 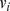 is the rate as a function of the concentrations and system parameters, and 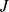 and 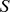 are steady-state fluxes and concentrations as functions of the system parameters. Control is a distributed systemic property. If there is a change in control at one point, it will be compensated for elsewhere in the network. The reactions that exert the largest control can be expected to define the behaviour of the system most heavily. Further information can be found in Heinrich and Schuster [39]. We define the general flux control exerted by reaction 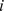 by the root sum of squares of all its flux control coefficients



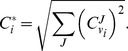
(5)The 5% of reactions with the highest 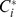 were considered high-control reactions.

## Supporting Information

File S1 **Main supplementary text that includes tables of all data included in the models, figures of simulations for the models, and extra notes on method implementation.**
(PDF)

File S2 **SBML file containing the yeast model, built without regulatory information.**
(XML)

File S3 **SBML file containing the yeast model, built with regulatory information.**
(XML)

File S4 **Excel file containing detailed steady state fluxes and concentrations obtained from the primary model (File S2).**
(XLSX)

## References

[1] Li C, Donizelli M, Rodriguez N, Dharuri H, Endler L, et al (2010) BioModels Database: An enhanced, curated and annotated resource for published quantitative kinetic models. BMC Systems Biology 4: 92. 20587024 10.1186/1752-0509-4-92 PMC2909940

[2] Herrgård MJ, Swainston N, Dobson P, Dunn WB, Arga KY, et al (2008) A consensus yeast metabolic network reconstruction obtained from a community approach to systems biology. Nature Biotechnology 26: 1155–60. 10.1038/nbt1492 PMC4018421 18846089

[3] Dobson PD, Smallbone K, Jameson D, Simeonidis E, Lanthaler K, et al (2010) Further developments towards a genome-scale metabolic model of yeast. BMC Systems Biology 4: 145. 21029416 10.1186/1752-0509-4-145 PMC2988745

[4] Schuster S (1999) Detection of elementary flux modes in biochemical networks: a promising tool for pathway analysis and metabolic engineering. Trends in Biotechnology 17: 53–60. 10087604 10.1016/s0167-7799(98)01290-6

[5] Orth JD, Thiele I, Palsson Bø (2010) What is flux balance analysis? Nature biotechnology 28: 245–248. 10.1038/nbt.1614 PMC3108565 20212490

[6] Mendes P, Stanford NJ, Smallbone K (2011) Kinetic modelling of large-scale metabolic networks. In: Proceedings of the 9th International Conference on Computational Methods in Systems Biology. ACM, pp. 5–6.

[7] Wang L, Birol I, Hatzimanikatis V (2004) Metabolic control analysis under uncertainty: framework development and case studies. Biophysical Journal 87: 3750. 15465856 10.1529/biophysj.104.048090 PMC1304888

[8] Wang L, Hatzimanikatis V (2006) Metabolic engineering under uncertainty. I: framework development. Metabolic Engineering 8: 133. 16414298 10.1016/j.ymben.2005.11.003

[9] Miskovic L, Hatzimanikatis V (2010) Production of biofuels and biochemicals: in need of an oracle. Trends in Biotechnology 28: 391–397. 20646768 10.1016/j.tibtech.2010.05.003

[10] Soh K, Miskovic L, Hatzimanikatis V (2012) From network models to network responses: integration of thermodynamic and kinetic properties of yeast genome-scale metabolic networks. FEMS Yeast Research. 10.1111/j.1567-1364.2011.00771.x 22129227

[11] Vargas FA, Pizarro F, Perez-Correa JR, Agosin E (2011) Expanding a dynamic flux balance model of yeast fermentation to genome-scale. BMC Systems Biology 5: 75. 21595919 10.1186/1752-0509-5-75 PMC3118138

[12] Steuer R, Gross T, Selbig J, Blasius B (2006) Structural kinetic modeling of metabolic networks. Proc Natl Acad Sci USA 103: 11868–11873. 16880395 10.1073/pnas.0600013103 PMC1524928

[13] Grimbs S, Selbig J, Bulik S, Holzhütter HG, Steuer R (2007) The stability and robustness of metabolic states: identifying stabilizing sites in metabolic networks. Molecular Systems Biology 3: 146. 18004279 10.1038/msb4100186 PMC2132447

[14] Smallbone K, Stanford NJ (2013) Kinetic modeling of metabolic pathways: Application to serine biosynthesis. In: Systems Metabolic Engineering, Humana Press. pp. 113–121. 10.1007/978-1-62703-299-5_7 23417802

[15] Teusink B, Walsh MC, van Dam K, Westerhoff HV (1998) The danger of metabolic pathways with turbo design. Trends in Biochemical Sciences 23: 162–169. 9612078 10.1016/s0968-0004(98)01205-5

[16] Savageau MA (1976) Biochemical Systems Analysis: A Study of Function and Design in Molecular Biology. Addison-Wesley Pub (Sd), 199 pp.

[17] Heijnen JJ (2005) Approximative kinetic formats used in metabolic network modeling. Biotechnology and Bioengineering 91: 534–45. 16003779 10.1002/bit.20558

[18] Liebermeister W, Klipp E (2006) Bringing metabolic networks to life: convenience rate law and thermodynamic constraints. Theor Biol Med Model 3: 41. 17173669 10.1186/1742-4682-3-41 PMC1781438

[19] Ederer M, Gilles ED (2007) Thermodynamically feasible kinetic models of reaction networks. Biophysical Journal 92: 1846–57. 17208985 10.1529/biophysj.106.094094 PMC1861785

[20] Liebermeister W, Uhlendorf J, Klipp E (2010) Modular rate laws for enzymatic reactions: thermodynamics, elasticities, and implementation. Bioinformatics 26: 1528–1534. 20385728 10.1093/bioinformatics/btq141

[21] Smallbone K, Simeonidis E, Swainston N, Mendes P (2010) Towards a genome-scale kinetic model of cellular metabolism. BMC Systems Biology 4: 6. 20109182 10.1186/1752-0509-4-6 PMC2829494

[22] Smallbone K, Simeonidis E, Broomhead DS, Kell DB (2007) Something from nothing: bridging the gap between constraint-based and kinetic modelling. The FEBS Journal 274: 5576–85. 17922843 10.1111/j.1742-4658.2007.06076.x

[23] Li P, Dada JO, Jameson D, Spasic I, Swainston N, et al (2010) Systematic integration of experimental data and models in systems biology. BMC Bioinformatics 11: 582. 21114840 10.1186/1471-2105-11-582 PMC3008707

[24] Rojas I, Golebiewski M, Kania R, Krebs O, Mir S, et al (2007) SABIO-RK: a database for biochemical reactions and their kinetics. BMC Systems Biology 1: S6.

[25] Lubitz T, Schulz M, Klipp E, Liebermeister W (2010) Parameter balancing for kinetic models of cell metabolism. J Phys Chem B 114: 16298–16303. 21038890 10.1021/jp108764b PMC2999964

[26] Gutenkunst R, Waterfall J, Casey F, Brown K, Myers C, et al (2007) Universally sloppy parameter sensitivities in systems biology models. PLoS Computational Biology 3: e189. 10.1371/journal.pcbi.0030189 PMC2000971 17922568

[27] Heinemann M, Sauer U (2010) Systems biology of microbial metabolism. Current opinion in microbiology 13: 337–343. 20219420 10.1016/j.mib.2010.02.005

[28] Wegscheider R (1902) Über simultane Gleichgewichte und die Beziehungen zwischen Thermodynamik und Reactionskinetik homogener Systeme. Z Phys Chem 39: 257–303.

[29] Haldane J (1930) Enzymes. Longmans, Green and Co., London. (republished in 1965 by MIT Press, Cambridge, MA).

[30] Kholodenko B, Cascante M, Hoek J, Westerhoff H, Schwaber J (2000) Metabolic design: how to engineer a living cell to desired metabolite concentrations and fluxes. Biotechnology and Bioengineering 59: 239–247. 10.1002/(sici)1097-0290(19980720)59:2<239::aid-bit11>3.0.co;2-9 10099334

[31] Swainston N, Smallbone K, Mendes P, Kell D, Paton N (2011) The SuBliMinaL Toolbox: automating steps in the reconstruction of metabolic networks. Journal of Integrative Bioinformatics 8: 186. 22095399 10.2390/biecoll-jib-2011-186

[32] Smallbone K, Simeonidis E (2009) Flux balance analysis: a geometric perspective. Journal of Theoretical Biology 258: 311–315. 19490860 10.1016/j.jtbi.2009.01.027

[33] Thomas S, Fell DA (1998) A control analysis exploration of the role of ATP utilisation in glycolytic-flux control and glycolytic-metabolite-concentration regulation. European Journal of Biochemistry 258: 956–67. 9990313 10.1046/j.1432-1327.1998.2580956.x

[34] Oehlen LJWM, Scholte ME, de Koning W, van Dam K (1994) Decrease in glycolytic flux in *Saccharomyces cerevisiae* cdc35–1 cells at restrictive temperature correlates with a decrease in glucose transport. Microbiology 140: 1891–1898. 7921242 10.1099/13500872-140-8-1891

[35] Bakker BM, Westerhoff HV, Opperdoes FR, Michels PA (2000) Metabolic control analysis of glycolysis in trypanosomes as an approach to improve selectivity and effectiveness of drugs. Molecular and Biochemical Parasitology 106: 1–10. 10743606 10.1016/s0166-6851(99)00197-8

[36] Reijenga KA, Snoep JL, Diderich JA, van Verseveld HW, Westerhoff HV, et al (2001) Control of glycolytic dynamics by hexose transport in *Saccharomyces cerevisiae* . Biophysical Journal 80: 626–34. 11159431 10.1016/S0006-3495(01)76043-2 PMC1301262

[37] Korzeniewski B (1998) Regulation of ATP supply during muscle contraction: theoretical studies. Biochemical Journal 330 (Pt 3: 1189–95. 10.1042/bj3301189 PMC1219260 9494084

[38] Somsen OJ, Hoeben MA, Esgalhado E, Snoep JL, Visser D, et al (2000) Glucose and the ATP paradox in yeast. Biochemical Journal 352 Pt 2: 593–9. PMC1221493 11085955

[39] Heinrich R, Schuster S (1996) The regulation of cellular systems, volume 416. Chapman & Hall New York.

[40] Hucka M, Finney A, Sauro H, Bolouri H, Doyle J, et al (2003) The systems biology markup language (sbml): a medium for representation and exchange of biochemical network models. Bioinformatics 19: 524–531. 12611808 10.1093/bioinformatics/btg015

[41] Hoops S, Sahle S, Gauges R, Lee C, Pahle J, et al (2006) Copasi: a complex pathway simulator. Bioinformatics 22: 3067–3074. 17032683 10.1093/bioinformatics/btl485

[42] Sanner M (1999) Python: a programming language for software integration and development. J Mol Graph Model 17: 57–61. 10660911

[43] Hynne F, Dan S, Srensen P (2001) Full-scale model of glycolysis in saccharomyces cerevisiae. Biophysical Chemistry 94: 121–163. 11744196 10.1016/s0301-4622(01)00229-0

[44] Teusink B, Passarge J, Reijenga CA, Esgalhado E, van Der Weijden CC, et al (2000) Can yeast glycolysis be understood in terms of in vitro kinetics of the constituent enzymes? Testing biochemistry. European Journal of Biochemistry 267: 5313–29. 10951190 10.1046/j.1432-1327.2000.01527.x

[45] Pritchard L, Kell DB (2002) Schemes of flux control in a model of *Saccharomyces cerevisiae* glycolysis. European Journal of Biochemistry 269: 3894–3904. 12180966 10.1046/j.1432-1033.2002.03055.x

[46] Conant GC, Wolfe KH (2007) Increased glycolytic flux as an outcome of whole-genome duplication in yeast. Molecular Systems Biology 3: 129. 17667951 10.1038/msb4100170 PMC1943425

[47] Scheer M, Grote A, Chang A, Schomburg I, Munaretto C, et al (2010) BRENDA, the enzyme information system in 2011. Nucleic Acids Research 39: D670–D676. 21062828 10.1093/nar/gkq1089 PMC3013686

