# Supplementary material for: Systematic Construction of Kinetic Models from Genome-Scale Metabolic Networks
Source: PLoS One. 2013 Nov 14;8(11):e79195. doi: 10.1371/journal.pone.0079195 (PMC3852239; doi:10.1371/journal.pone.0079195)
Supplement: File S1 — Main supplementary text that includes tables of all data included in the models, figures of simulations for the models, and extra notes on method implementation. (PDF) [file pone.0079195.s001.pdf]

Systematic Construction of Kinetic Models from Genome-Scale Metabolic Networks

Supplementary Text and Tables

Natalie J. Stanford,Timo Lubitz, Kieran Smallbone,
Edda Klipp, Pedro Mendes, Wolfram Liebermeister

**Table 1**	KEGG pathways represented in the yeast kinetic model
**Table 2**	Steady state fluxes from models in BioModels Database
**Table 3**	Yeast specific intracellular metabolite concentrations
**Table 4**	Extracellular metabolite concentrations
**Table 5**	Equilibrium constants taken from models in BioModels Database
**Table 6**	Extracellular metabolite concentrations manually adjusted
**Table 7**	Allosteric regulators
**Figure A**	Fluxes after an increase in extracellular glucose concentration
**Figure B**	Fluxes after a decrease in extracellular glucose concentration
**Figure C**	Concentrations after an increase in extracellular glucose concentration
**Figure D**	Concentrations after a decrease in extracellular glucose concentration
**Figure E**	Glycolysis concentrations after an increase in extracellular glucose concentration
**Figure F**	Glycolysis concentrations after a decrease in extracellular glucose concentration
**Figure G**	Scaled flux control coefficients in the yeast metabolic model
**Figure H**	Control Patterns within the regulation model
**Note i**	Important aspects of method implementation
**Note ii**	Checking flux distributions for thermodynamic feasibility

Table 1:
KEGG pathways represented in the yeast kinetic model

Pathway	Number of reactions
Purine and pyrimidine biosynthesis	27
Lipid metabolism	19
Valine, leucine, and isoleucine biosynthesis	17
Glycolysis / gluconeogenesis	16
Nucleotide salvage pathway	15
Tyrosine, tryptophan, and phenylalanine metabolism	15
Arginine and proline metabolism	12
Carbohydrate and lipid metabolism	11
Histidine metabolism	10
Biosynthesis of secondary metabolites	9
Cofactor and prosthetic group biosynthesis	9
Alternate carbon metabolism	8
Transport, outer membrane porin	8
Citric acid cycle	7
Glycerophospholipid metabolism	7
Glycerolipid metabolism	5
Membrane lipid metabolism	5
Pentose phosphate pathway	5
Threonine and lysine metabolism	5
Biosynthesis of unsaturated fatty acids	4
Cell envelope biosynthesis	4
Cysteine and methionine metabolism	4
Glycine and serine metabolism	4
Lysine biosynthesis	4
Oxidative phosphorylation	4
Alanine and aspartate metabolism	3
Anaplerotic reactions	3
Sulfur metabolism	3
Alanine, aspartate, and glutamate metabolism	2
Folate metabolism	2
Methionine metabolism	2
N-glycan biosynthesis	2
Pentose and glucuronate interconversions	2
Riboflavin metabolism	2
Starch and sucrose metabolism	2
Miscelleneous	28

The table lists metabolic pathways (as defined in the KEGG database) covered by the yeast kinetic model. For this table, reactions were uniquely assigned to one of the pathways.

Table 2:
Steady state fluxes calculated from models 61, 64, 172, 176, and 177 in BioModels Database

Reaction	Assigned flux (mM/s)	Model flux (mM/s)
alcohol dehydrogenase, reverse reaction (acetaldehyde -> ethanol)	1.17	8.19 $\times 10^{-1}$
ATPase, cytosolic	5.95 $\times 10^{-1}$	4.17 $\times 10^{-1}$
enolase	1.76	1.23
fructose-bisphosphate aldolase	7.33 $\times 10^{-1}$	6.23 $\times 10^{-1}$
glucose-6-phosphate isomerase	7.33 $\times 10^{-1}$	5.99 $\times 10^{-1}$
glyceraldehyde-3-phosphate dehydrogenase	1.06	1.23
glycerol-3-phosphatase	5.10 $\times 10^{-2}$	4.53 $\times 10^{-2}$
glycerol-3-phosphate dehydrogenase (NAD)	1.49 $\times 10^{-1}$	1.04 $\times 10^{-1}$
hexokinase (D-glucose:ATP)	8.66 $\times 10^{-1}$	1.81 $\times 10^{-1}$
phosphofructokinase	6.06 $\times 10^{-1}$	6.23 $\times 10^{-1}$
phosphoglycerate kinase	8.75 $\times 10^{-1}$	1.23
phosphoglycerate mutase	1.76	1.23
pyruvate decarboxylase	1.25	8.18$\times 10^{-1}$
pyruvate kinase	1.06	1.21
triose-phosphate isomerase	3.95 $\times 10^{-1}$	6.23$\times 10^{-1}$

To obtain a set of flux data, we selected a group of metabolic models from BioModels Database [5] that are yeast-specific and contain glucose as the primary carbon source (models 61, 64, 172, 176, and 177). Each model was run to a steady state from its operating state and the resulting flux for each reaction was noted. Where more than one model provided flux values for the same reaction, the median value was used.

Table 3:
Yeast specific intracellular metabolite concentrations taken from models 61, 64, 172, 176, and 177 in BioModels Database

Intracellular metabolite	Concentration (mM)
2-phospho-D-glyceric acid	5.15 $\times$ 10 $^{-2}$
3-phospho-D-glyceric acid	3.63 $\times$ 10 $^{-1}$
3-phospho-D-glyceroyl dihydrogen phosphate	1.09 $\times$ 10 $^{-4}$
acetaldehyde	1.20 $\times$ 10 $^{-1}$
AMP	1.26
ATP	1.09
ADP	1.72
beta-D-glucose 6-phosphate	4.96 $\times$ 10 $^{-1}$
carbon dioxide	1.00
D-fructose 1,6-bisphosphate	1.34
D-fructose 6-phosphate	1.05 $\times$ 10 $^{-1}$
D-glucose	9.88 $\times$ 10 $^{-2}$
ethanol	5.00 $\times$ 10 $^{+1}$
glyceraldehyde 3-phosphate	4.36 $\times$ 10 $^{-2}$
glycerol	1.50 $\times$ 10 $^{-1}$
glycerone phosphate	6.02 $\times$ 10 $^{-1}$
NAD(+)	1.50
NADH	8.67 $\times$ 10 $^{-2}$
phosphoenolpyruvate	2.71 $\times$ 10 $^{-2}$
pyruvate	6.06 $\times$ 10 $^{-2}$
sn-glycerol 3-phosphate	1.29 $\times$ 10 $^{+1}$

The intracellular metabolite concentrations were taken from yeast-specific models in BioModels Database that use glucose as the primary carbon source. Where concentrations were not known, the intracellular concentrations were taken to be the median value of the intracellular values, 0.549 mM.

Table 4:
Extracellular metabolite concentrations taken from yeast footprinting media

Extracellular metabolite	Concentration (mM)
ammonium	3.80$\times 10^{+1}$
D-glucose	1.11$\times 10^{+1}$
glycerol	1.76
sulphate	4.22 $\times 10^{+1}$
succinate$^{(2-)}$	1

The extracellular metabolites were taken from yeast-specific models in BioModels Database that use glucose as the primary carbon source. For unknown extracellular values median value of all extracellular concentrations was used (24.5 mM).

Table 5:
Equilibrium constants taken from models in BioModels Database.

Reaction	Equilibrium constant
hexokinase (D-glucose:ATP) [glucose $\rightarrow$ ...]	2.00$\times 10^{3}$
glucose-6-phosphate isomerase [glucose-6-p. $\rightarrow$ ...]	2.90$\times 10^{-1}$
fructose-bisphosphate aldolase [fructose-1,6-bisphosphate $\rightarrow$ ...]	4.50$\times 10^{-1}$
glyceraldehyde-3-phosphate dehydrogenase [glyceraldehyde-3-p. $\rightarrow$ ...]	3.20$\times 10^{3}$
phosphoglycerate mutase [3-phosphoglycerate $\rightarrow$ ...]	6.70
enolase [2-phosphoglycerate $\rightarrow$ ...]	6.50$\times 10^{3}$

Where appropriate, equilibrium constants were taken from models available in BioModels Database that use glucose as their primary carbon source (models 61, 64, 172, 176, and 177). All transport reactions were set to have equilibrium constants of 1.

Table 6:
Extracellular metabolite concentrations manually adjusted

Extracellular metabolite	Concentration (mM)
carbon dioxide	0

Transport reactions were set to have a fixed equilibrium value of 1 because the metabolite is only being moved across a membrane. For export reactions assigned with the median extracellular concentration value this caused problems with the reaction directionality. The above concentrations were adjusted to ensure that the behaviour of the cell was consistent with the flux data. Only concentrations computed using the approximation values were allowed to be adjusted.

Table 7:
Allosteric Regulators

Reaction	Species	Value
glyceraldehyde-3-phosphate dehydrogenase	glyceraldehyde-3-phosphate	0.0012
phosphoglycerate mutase	2-phospho-D-glyceric acid	0.8
pyruvate decarboxylase	pyruvate	2.75
phosphoglycerate kinase	ATP	0.525
glucose-6-phosphate isomerase	D-glucose	0.7
glucose-6-phosphate isomerase	beta-D-glucose-6-phosphate	0.7
phosphofructokinase	ATP	4
alpha-alpha trehalose-phosphate synthase	UDP-glucose	6

Known inhibition constants taken from the BRENDA database

.

Figure A:
Fluxes after an increase in extracellular glucose concentration

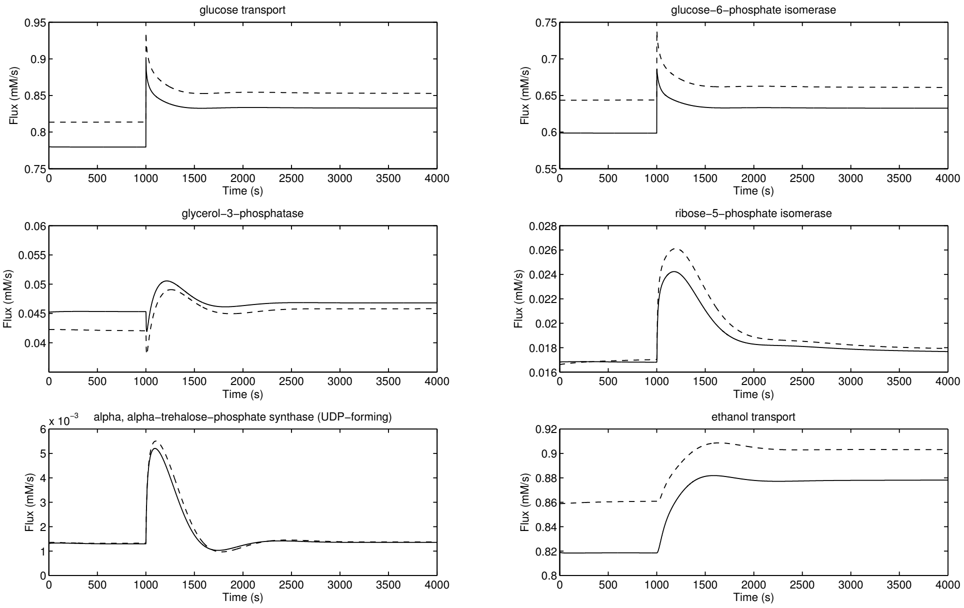

Flux changes after an increase in extracellular glucose for a selection of reactions within the central carbon metabolism. These provide an indication of behaviour around key points of branching. (–) standard model; (- -) regulation model.

Figure B:
Fluxes after a decrease in extracellular glucose concentration

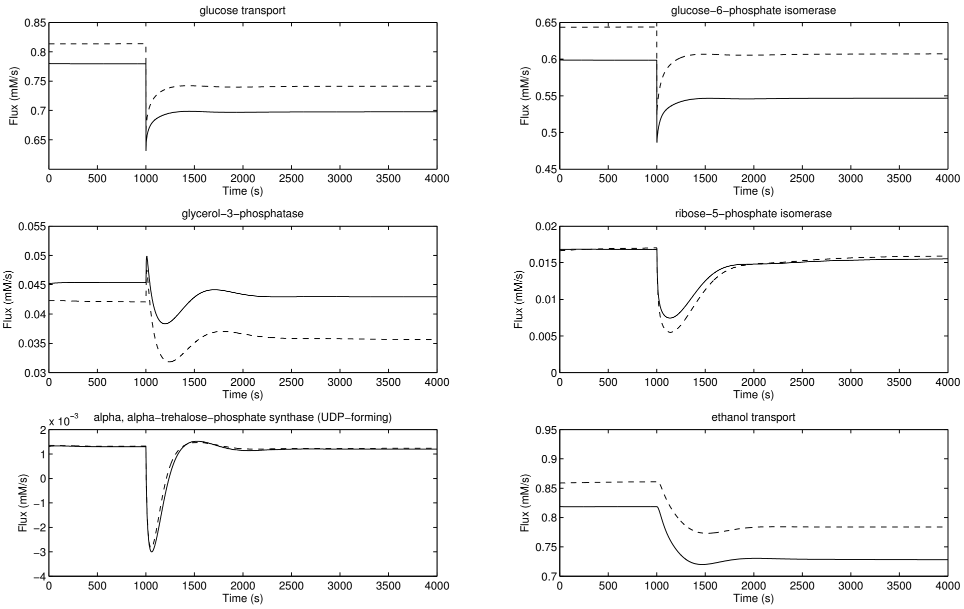

Flux changes after a decrease in extracellular glucose for a selection of reactions within the central carbon metabolism. These provide an indication of behaviour around key points of branching. (–) standard model; (- -) regulation model.

Figure C:
Concentrations after an increase in extracellular glucose concentration

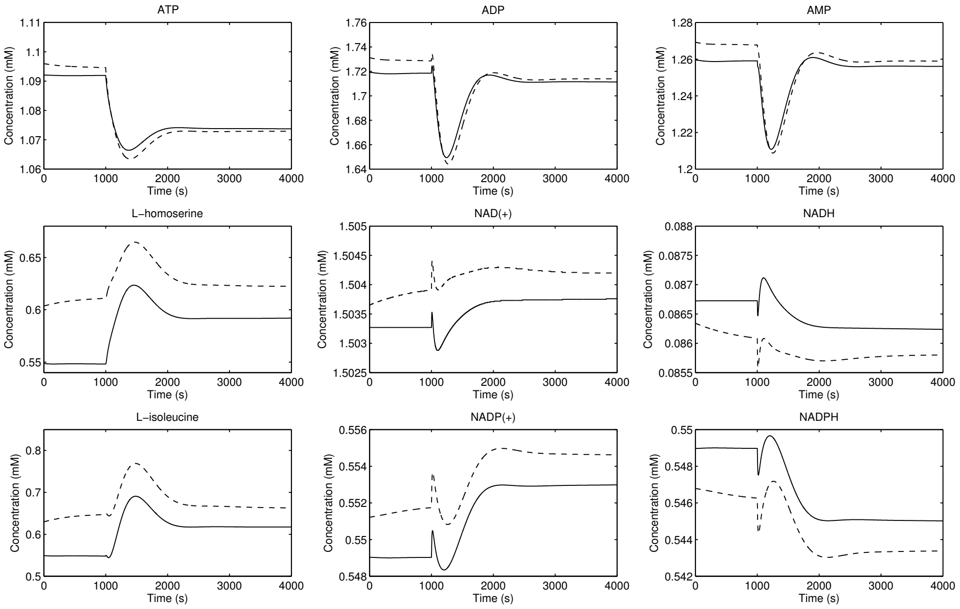

Concentration changes after an increase in extracellular glucose, in the balances of ATP/ADP/AMP, NAD/NADH, and NADP/NADPH. Amino acids isoleucine and homoserine are also shown. (–) standard model; (- -) regulation model.

Figure D:
Concentrations after a decrease in extracellular glucose concentration

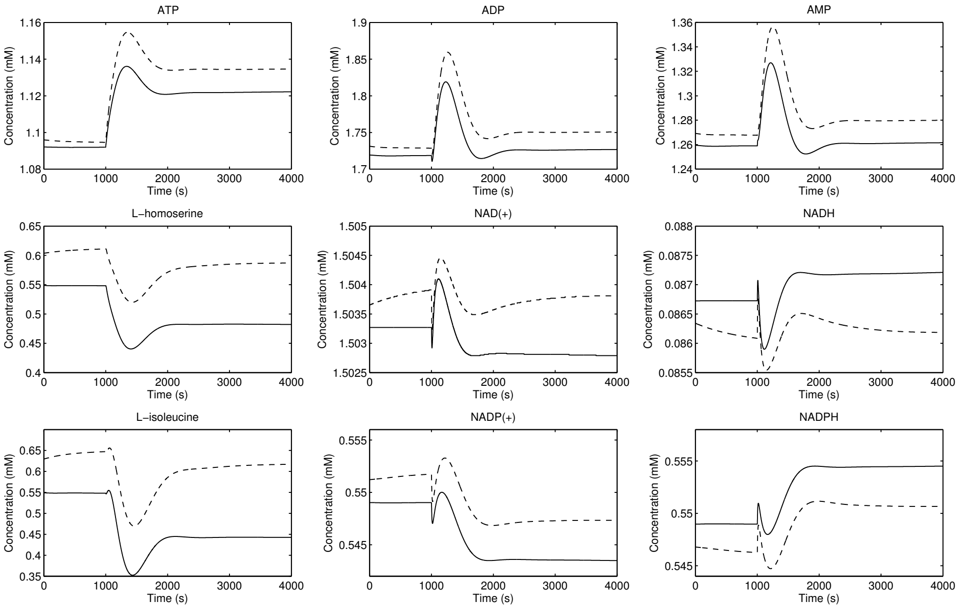

Concentration changes after a decrease in extracellular glucose, in the balances of ATP/ADP/AMP, NAD/NADH, and NADP/NADPH. Amino acids isoleucine and homoserine are also shown. (–) standard model; (- -) regulation model.

Figure E:
Concentrations in glycolysis after an increase in extracellular glucose concentration

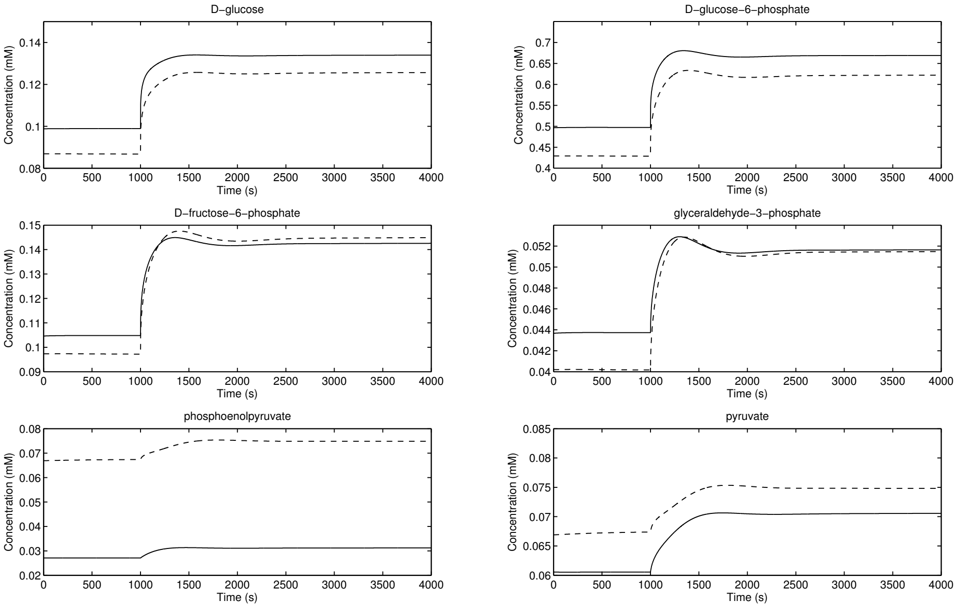

Concentration changes of glycolytic metabolites after an increase in extracellular glucose. These are located at key branching points along the glycolysis network and give an indication of short term intracellular pool responses to extracellular glucose perturbations. (–) standard model; (- -) regulation model.

Figure F:
Concentrations in glycolysis after a decrease in extracellular glucose concentration

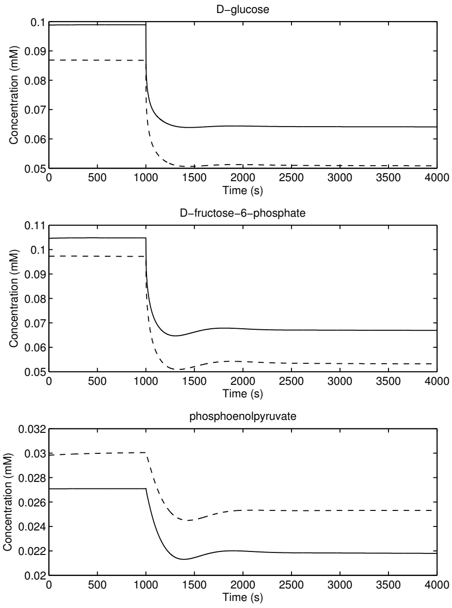

Concentration changes of glycolytic metabolites after a decrease in extracellular glucose. These are located at key branching points along the glycolysis network and give an indication of short term intracellular pool responses to extracellular glucose perturbations. (–) standard model; (- -) regulation model.

Figure G:
Scaled flux control coefficients in the yeast metabolic model

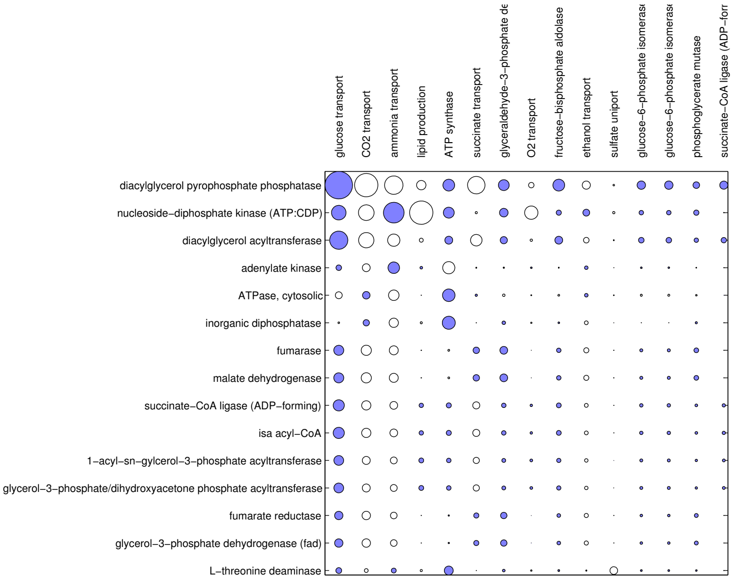

Figure 1: Scaled flux control coefficients in the yeast metabolic model (compare Figure 3B in article). Absolute values are shown by circle areas, blue and white circles represent positive and negative values, respectively. Rows and columns correspond to the controlled and controlling reactions. The reactions were sorted by overall (sum of square) control coefficients; only the fifteen highest-ranking reactions are shown, respectively, for rows and columns.

Figure H:
Control Patterns within the regulation model

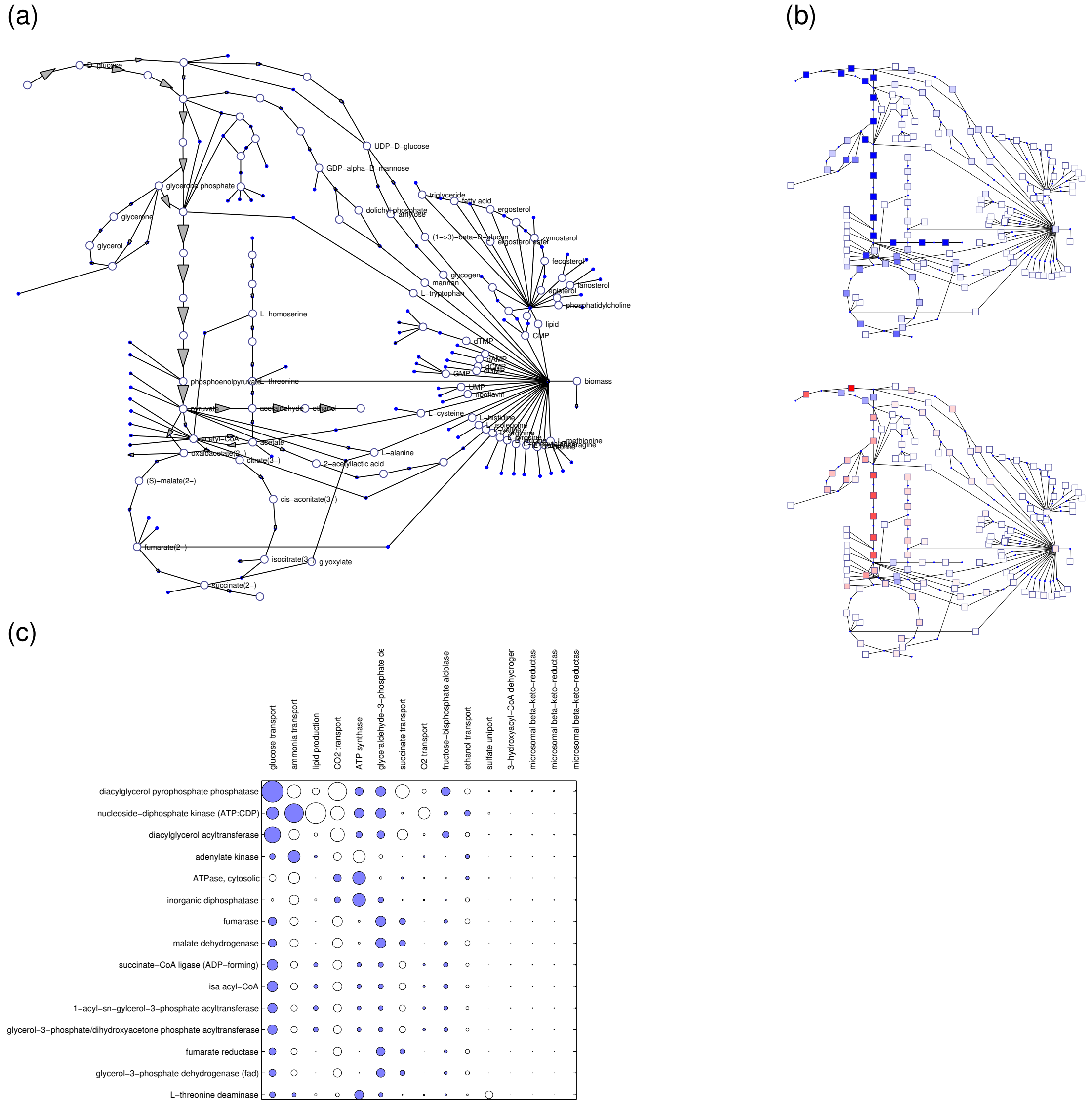

Figure 2: Fluxes and control coefficients in the yeast metabolic model with allosteric regulation (compare Figure 3 in article). (a) Fluxes obtained from Geometric FBA. Only selected reactions with large fluxes are depicted, co-substrates are not shown (flux directions and magnitudes shown by arrows). (b) Control coefficients. Top: control exerted by the glucose transporter (GluT). Unscaled flux control coefficients are shown in shades of blue (positive values) and red (negative values). Bottom: control exerted by the biomass production reaction. High-flux reactions respond most strongly: an increased glucose import increases the glycolytic flux, while increased biomass production directs fluxes to other pathways and thereby decreases the glycolytic flux. (c) Scaled flux control coefficients in the yeast metabolic model. Absolute values are shown by circle areas, blue and white circles represent positive and negative values, respectively. Rows and columns correspond to the controlled and controlling reactions. The reactions were sorted by overall (sum of square) control coefficients; only the fifteen highest-ranking reactions are shown, respectively, for rows and columns.

Note i:
Aspects of implementation, method extensions, and alternative approaches to data integration

(a) Data input and output Models are most frequently constructed to answer specific biological questions, and they refer to certain experimental conditions or states of the cell, which limits their validity to these conditions. As such, **the fluxes** must only be taken from bottom-up models that are valid under the exact conditions that will be investigated using the final model, and reflect only a single set of external conditions which influence the steady state. The flux data should include information about reactions that cannot carry any flux within the organism (e.g. where there is a strain-specific gene-knockout), and should also include broad reaction directionality for uptake reactions (e.g. the cell must import oxygen, so the reaction `oxygen [ex]` → `oxygen [in]` must hold a positive flux). Flux data taken from experiments performed under the investigable growth conditions can also be used.

For the generation of a more specific large-scale metabolic model, it would be advised that the **metabolite concentrations** are collected using metabolomic quantification and footprinting techniques that are specific to the desired organism and biological state. Where more metabolite data are available, it may also be possible to use thermodynamic FBA to help improve the estimated values of metabolites with unknown concentrations [3]. The assignment of many of the metabolite concentration values, through their relationship with the mass-action ratios, have a direct impact on the bounds for computing the equilibrium constants, and therefore the favoured direction of the transport reactions. If these produce flux directions that are contrary to known network behaviour (either from models or experimental knowledge) or where they cause issues with equilibrium fitting, they should be altered to more accurately reflect the known system behaviour.

Regarding **kinetic and thermodynamic constants**, only a small number of equilibrium constants are known from models, and some extra data can be found in the literature. Coupled to this there are some methods such as 'eQuilibrator' [2] which can allow for the computation of potential equilibrium constants based on the structural properties of the metabolites associated with a given reaction, and corresponding Gibbs free energies. These can be used, but we would recommend reflecting "confidence" levels in the values with their bounds during balancing: that is, correctly calculated data or constants taken from models are fixed, and other data have increasing boundary sizes as confidence in the values reduces. It is important to note that the values of all equilibrium constants must be above the mass action ratio for the reaction.

(b) Steps of the workflow For computing the flux solution, also other **FBA methods** could be used, which are described in [1] or [4], as well as other objective functions in conjunction with flux data. When **restricting the full network to a network of interest**, it is important to consider what the model will be used for: retention of all important pathways to the investigation is important. The efficiency of **parameter balancing** is upmost dependent on the quality of the input parameters. The less confidence there is in the origin of the parameters (i.e. heterogenous sources or measuring conditions) the broader the corresponding standard deviations have to be set in order to not bias the outcome of the posterior distribution. On the contrary, if certain parameters need to be kept very close to the input value, either the standard deviation can be chosen very tightly, or inequality constraints can be set for the optimisation process. The latter will allow the value to move between lower and upper bounds, but not beyond. For the optimisation process an algorithm of convex minimisation is sufficient.

The initial construction of the model requires that all **rate laws** take a generic form as above, but it is possible to vary the mechanistic behaviour of the reactions to reflect behaviour that may be more appropriate to different organisms. *In vitro* measured kinetic rate laws can be substituted in if desired, but this is recommended *after* the model has been scaled to the correct steady state

(Step 5). To insert the rate law, we need to ensure that it matches the model in its equilibrium constant, its mass-action ration, and its reaction rate. The first two quantities have to be imposed on the network model beforehand, during its construction.

Adjust model to steady-state flux. The correct rate can be obtained by adjusting the maximal velocity in the rate law. We recommend that alterations are again kept to scaling of V_{m} (or to scaling of enzyme concentrations, if the maximal velocities V_{m} are represented as products of enzyme concentrations and catalytic constants). If the network is not reduced in earlier steps there will be a large number of inactive reactions, which must be set to 0 manually. The reactions should be set to 0 during this step, but perhaps with modifications that 'activate' V_{m} to a positive value under certain conditions. **Metabolic control analysis** reflects only the current state of the system. Once a reaction is identified as having a large flux control, it can be replaced by an *in vitro* calculated rate law for that reaction. If a rate law substitution is made (or parameters are altered owing to new information) then the MCA should be recalculated so a new reflection of the steady state is included.

Note ii:
Checking flux distributions for thermodynamic feasibility

A thermodynamically feasible flux distribution has to agree with the chemical potential differences in the network. For ideal chemical mixtures, the chemical potentials μ_i can be expressed in terms of the concentrations c_i and the chemical potentials $\mu_i^{(0)}$ at standard concentration $c = 1$ mM as

$$\mu_i = \mu^{(0)} + RT \ln c_i. \tag{1}$$

The reaction affinity – defined as the negative reaction Gibbs free energy – is given by

$$A_l = -\Delta\mu_l = -\sum_i n_{il}\, \mu_i. \tag{2}$$

The reaction affinity determines the flux direction: flux and affinity need to have the same sign unless the flux vanishes. Therefore, to ensure that a flux distribution is thermodynamically feasible, we need to check whether there exists a set of chemical potentials μ_i such that

$$v_l \neq 0 \qquad \Rightarrow \qquad \text{sign}(v_l) = \text{sign}(A_l) \tag{3}$$

for all reactions l. This can be checked by linear programming, as we will explain now. By inserting Eq. (1), the reaction affinities can be written in terms of concentrations by

$$\begin{aligned} A_l &= -\sum_i n_{il}\, \mu_i^{(0)} - \sum_i n_{il}\, RT \ln c_i = RT\, \ln K_{\text{eq},l} - RT \sum_i n_{il}\, \ln c_i \\ &= RT \ln\left(\frac{K_{\text{eq},l}}{\prod_i c_i^{n_{il}}}\right) \end{aligned} \tag{4}$$

Thermodynamics requires that a non-zero reaction rate has the same sign as the corresponding reaction affinity. However, if reactions are close to equilibrium ($A \approx 0$), but show a finite flux, a large enzyme level may be required. To avoid extreme enzyme demands, we may require reactions with non-zero fluxes to show reaction affinities above some threshold value, $|A_l| \geq A_{\min}$. One possibility to choose $A_{\min}$ is by predefining the corresponding ratio of forward and reverse rates, given by

$$\ln \frac{v_l^+}{v_l^-} = \frac{A}{RT} \tag{5}$$

Postulating that the forward flux v_l^+ must not exceed the (positive) net flux by more than a 100-fold, we obtain the condition

$$\begin{aligned} 10 &\geq \frac{v_l^+}{v_l^+ - v_l^-} \\ \Rightarrow \quad \frac{1}{10} \leq 1 - \frac{v_l^-}{v_l^+} &= 1 - \exp\left(-\frac{A_l}{RT}\right) \\ \Rightarrow \quad -\frac{A_l}{RT} &\leq \ln(1 - \frac{1}{10}) \\ \Rightarrow \quad A_l &\geq -RT\,\ln(1-\frac{1}{10}) \approx RT\,\frac{1}{10} \approx 0.25\,\text{kJ/mol} \end{aligned} \tag{6}$$

With these formulae, it is easy to check a given flux distribution for thermodynamic correctness. If a set of concentrations and standard chemical potentials is given, we just have to compute the reaction affinities and see if they show the correct signs and exceed the necessary thresholds. If no information is given except for the flux distribution, we can check if there exists a vector of chemical potentials that agree with the fluxes. We can formulate this as a set of linear constraints on μ:

$$\begin{aligned} & \mu^{\min} \leq \mu \leq \mu^{\max} \\ v_l > 0 \Rightarrow \quad & -\sum_i n_{il}\,\mu_l > A_{\min} \\ v_l < 0 \Rightarrow \quad & \sum_i n_{il}\,\mu_l < -A_{\min} \end{aligned} \tag{7}$$

where low thresholds $\mu^{\min}$ and high thresholds $\mu^{\max}$ are chosen to bound the solution space. Standard linear programming can be used to check if these constraints can be satisfied.

References

[1] D. A. Beard, S. Liang, and H. Qian. Energy balance for analysis of complex metabolic networks. *Biophysical Journal*, 83(1):79–86, 2002.

[2] A. Flamholz, E. Noor, A. Bar-Even, and R. Milo. eQuilibrator–the biochemical thermodynamics calculator. *Nucleic acids research*, 40(Database issue):D770–5, Jan. 2012.

[3] C. S. Henry, L. J. Broadbelt, and V. Hatzimanikatis. Thermodynamics-based metabolic flux analysis. *Biophysical journal*, 92(5):1792–805, Mar. 2007.

[4] A. Hoppe, S. Hoffmann, and H. Holzhütter. Including metabolite concentrations into flux-balance analysis: Thermodynamic realizability as a constraint on flux distributions in metabolic networks. *BMC Syst. Biol*, 1(1):23, 2007.

[5] C. Li, M. Donizelli, N. Rodriguez, H. Dharuri, L. Endler, V. Chelliah, L. Li, E. He, A. Henry, M. I. Stefan, J. L. Snoep, M. Hucka, N. Le Novère, and C. Laibe. BioModels Database: An enhanced, curated and annotated resource for published quantitative kinetic models. *BMC Systems Biology*, 4:92, Jan. 2010.
